# Therapeutic Targeting of the Proinflammatory IL-6-JAK/STAT Signalling Pathways Responsible for Vascular Restenosis in Type 2 Diabetes Mellitus

**DOI:** 10.1155/2019/9846312

**Published:** 2019-01-02

**Authors:** Florah Tshepo Moshapa, Kirsten Riches-Suman, Timothy Martin Palmer

**Affiliations:** ^1^School of Pharmacy and Medical Sciences, Faculty of Life Sciences, University of Bradford, Bradford BD7 1DP, UK; ^2^School of Chemistry and Biosciences, Faculty of Life Sciences, University of Bradford, Bradford BD7 1DP, UK; ^3^Centre for Atherothrombosis and Metabolic Disease, Hull York Medical School, University of Hull, Hull HU6 7RX, UK

## Abstract

Type 2 diabetes mellitus (T2DM) is increasing worldwide, and it is associated with increased risk of coronary artery disease (CAD). For T2DM patients, the main surgical intervention for CAD is autologous saphenous vein grafting. However, T2DM patients have increased risk of saphenous vein graft failure (SVGF). While the mechanisms underlying increased risk of vascular disease in T2DM are not fully understood, hyperglycaemia, insulin resistance, and hyperinsulinaemia have been shown to contribute to microvascular damage, whereas clinical trials have reported limited effects of intensive glycaemic control in the management of macrovascular complications. This suggests that factors other than glucose exposure may be responsible for the macrovascular complications observed in T2DM. SVGF is characterised by neointimal hyperplasia (NIH) arising from endothelial cell (EC) dysfunction and uncontrolled migration and proliferation of vascular smooth muscle cells (SMCs). This is driven in part by proinflammatory cytokines released from the activated ECs and SMCs, particularly interleukin 6 (IL-6). IL-6 stimulation of the Janus kinase (JAK)/signal transducer and activator of transcription 3 (STAT) pathway is a key mechanism through which EC inflammation, SMC migration, and proliferation are controlled and whose activation might therefore be enhanced in patients with T2DM. In this review, we investigate how proinflammatory cytokines, particularly IL-6, contribute to vascular damage resulting in SVGF and how suppression of proinflammatory cytokine responses via targeting the JAK/STAT pathway could be exploited as a potential therapeutic strategy. These include the targeting of suppressor of cytokine signalling (SOCS3), which appears to play a key role in suppressing unwanted vascular inflammation, SMC migration, and proliferation.

## 1. Introduction

Diabetes mellitus (DM) is a group of metabolic disorders characterised by uncontrollable high blood glucose levels caused by defects in insulin action, insulin secretion, or both. The global prevalence of diabetes has increased from 4.7% in 1980 to 8.5% in 2014 (http://www.who.int/diabetes/global-report/en/), and in the UK alone, diagnosis of diabetes has more than doubled in the last 20 years with 3.7 million diagnosed patients in the UK in 2017 (https://www.diabetes.org.uk). Approximately 90% of these patients have type 2 diabetes mellitus (T2DM), which confers a 2–4 fold increased risk of developing cardiovascular disease (CVD) compared to nondiabetes mellitus (NDM) patients and is the leading cause of death in the diabetic population [1, 2].

Revascularisation using coronary artery bypass grafts (CABG) is frequently required in T2DM patients as the diffuse nature of their CVD is often multivessel [3], although it is unclear whether or not T2DM is associated with poorer outcomes following coronary revascularisation [4, 5]. Saphenous vein graft failure (SVGF) is a significant clinical problem with ∼50% of SV grafts completely occluded at 10 years, and an additional 25% with significant restenosis [6, 7]. The UK National Health Service (NHS) spends approximately £10 billion per year on the management of diabetes and its complications (https://www.diabetes.org.uk), thus there is a pressing need to develop innovative therapies to improve CABG outcome for diabetes patients.

T2DM patients are more susceptible to coronary artery disease (CAD) independently of glycaemic control [8]. However, while normalising blood glucose levels has been shown to reduce the incidence of microvascular complications such as diabetic nephropathy, neuropathy, and retinopathy [9, 10], there is no substantive evidence of any positive impact on macrovascular complications such as coronary artery disease, peripheral arterial disease, and stroke [11, 12]. This suggests that management of T2DM requires more than just glycaemic control and that other factors are involved in the development of macrovascular complications [13].

There is growing evidence that chronic vascular inflammation driven in part by proinflammatory cytokines can drive vascular complications of T2DM [14, 15]. Therefore, it is of interest to investigate how proinflammatory cytokines, particularly IL-6 which is elevated in T2DM, contribute to vascular damage resulting in SVGF and how suppression of proinflammatory cytokine responses via targeting the Janus kinase (JAK)/signal transducer and activator of transcription (STAT) pathway could be exploited as a potential therapeutic strategy.

## 2. Diabetes and Vein Graft Failure

CABG is a clinical mainstay for revascularisation and can be beneficial for patients with diabetes. A randomised clinical study of 300 T2DM patients indicated that CABG was associated with long survival rates (>10 years) and lower incidences of myocardial infarction as compared to percutaneous coronary intervention (PCI) procedures [16]. The autologous long SV is the most commonly used conduit due to its availability, length, and ease of harvesting. However, it displays lower long-term patency compared to grafts from the internal mammary artery (IMA) [6]. Reintervention as PCI or repeat-CABG are also associated with low survival rates and increased risk of other complications such as myocardial infarction and stroke. At 10 years, survival rates for repeat CABG patients are 55–75% and are associated with 4.7% increased incidence of stroke [17, 18].

SVGF occurs in three interlinked phases: thrombosis, neointimal hyperplasia (NIH), and accelerated atherosclerosis [19]. While thrombosis accounts for ∼12% of the incidences of vein graft failure that occur within 1 month postprocedure, atherosclerosis on NIH lesions accounts for 50% of late graft failures [6, 7]. Surgical technique, mechanical compression of the vein, anastomotic complications, and graft sepsis also contribute to restenosis [20].

**Figure 1:**
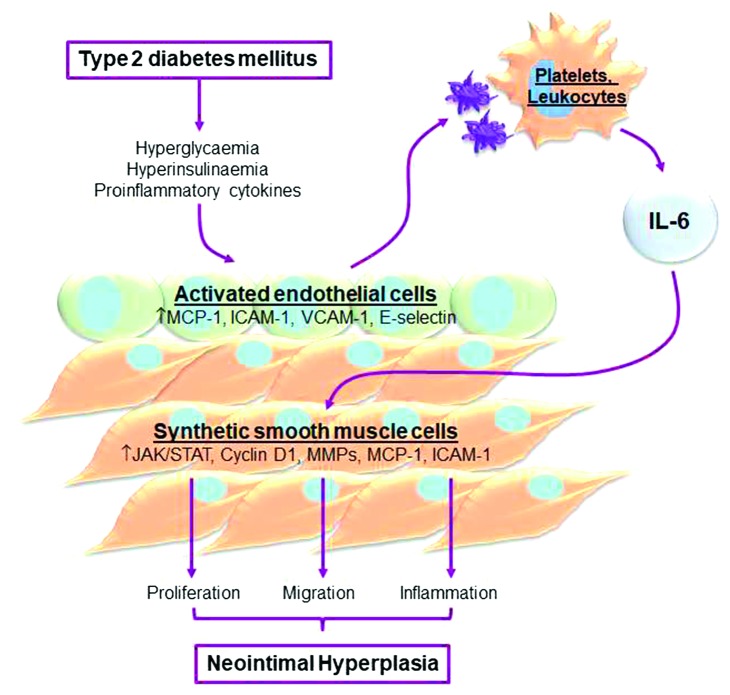
Cellular and molecular signals linking type 2 diabetes mellitus and neointimal hyperplasia. Type 2 diabetes is associated with circulating hyperglycaemia and hyperinsulinaemia and increased proinflammatory cytokines. Activated endothelial cells upregulate MCP-1, ICAM-1, VCAM-1, and E-selectin. This increases recruitment of platelets and leukocytes which release IL-6. This key cytokine acts on smooth muscle cells to activate JAK/STAT signalling to trigger upregulation of cyclin D1 and matrix metalloproteinases (MMPs) and further increase MCP-1 and ICAM-1 induction. These molecular signatures are characteristic of synthetic smooth muscle cells, leading to the development of neointimal hyperplasia.

The predominant cell type in the vascular wall is the smooth muscle cell (SMC), which is essential for maintenance of vascular tone through controlled contraction and relaxation. A single-cell thick layer of endothelial cells (EC) line the vessel lumen and communicate any changes in circulating factors or flow to the underlying SMCs [21]. The EC layer is often disrupted during the grafting procedure, leading to EC activation, and the exposure of underlying SMCs to increased pulsatile flow can also modulate phenotype. This leads to induction of adaptive physiological healing processes including vascular inflammation, SMC migration and proliferation, and secretion of matrix metalloproteinases (MMPs). However, these same processes can also trigger the development of pathological NIH, characterised by chronic vascular EC inflammation and uncontrollable SMC migration and proliferation (Figure 1) [22]. Importantly, human ECs and SMCs from T2DM patients already show defects in such cellular functions compared to cells from NDM patients [23–25]. NIH also narrows the blood vessel lumen and serves as a foundation for the development of atherosclerotic plaques, which contribute to SV restenosis [26]. These processes are partly regulated by proinflammatory cytokines such as IL-6, and high levels of IL-6 have been linked to post-CABG restenosis [27, 28].

EC activation is an early event in the development of NIH and thus a potential target to prevent SV restenosis [29]. Surgical manipulation (harvesting and implantation) and increased wall and shear stress during adaptation to the arterial circulation can damage and activate ECs [30, 31]. Activated ECs release proinflammatory cytokines and chemokines, e.g., monocyte chemotactic protein-1 (MCP-1), and upregulate adhesion molecules including platelet-endothelial cell adhesion molecule-1 (PECAM-1), intercellular adhesion molecule-1 (ICAM-1), vascular cell adhesion molecule-1 (VCAM-1), P-selectin, E-selectin, and subendothelial Von Willebrand factor (vWF) [32]. This leads to a cascade whereby activated platelets, macrophages, and SMCs further release bioactive molecules including proinflammatory cytokines such as IL-6 which stimulate SMC migration, proliferation, and expression of MMPs which degrade collagen and elastin, allowing SMC to invade the lumen (Figure 1) [19]. A study, specifically using HSV, demonstrated that MMP-2 activation and MMP-9 expression is increased in SV segments cultured for 14 days following surgical injury as compared to freshly excised veins [33]. Another study has shown that MMP-9 but not MMP-2 was upregulated in HSV organ culture (14 days) and coincided with the formation of NIH [34]. Consistent with a role for MMPs in driving NIH, adenovirus-mediated overexpression of tissue inhibitors of MMPs (TIMPs) isoforms significantly has been shown to reduce NIH in cultured HSV segments over 14 days and in a porcine model of vein graft failure for up to 3 months [35–37]. Persistence of a synthetic SMC phenotype leads to uncontrolled migration and proliferation and has been linked to development of pathological NIH [21, 38]. Conversely, an inability of SMCs to migrate and proliferate prevents adaptation and arterialisation of the vein conduit and has been linked to SVGF [24]. Thus, tight control of SMC phenotypic switching is a crucial determinant of vein graft patency.

### 2.1. Hyperglycaemia, Hyperinsulinaemia, and NIH

T2DM is characterised by hyperglycaemia and hyperinsulinaemia which can cause EC and SMC dysfunction either directly or indirectly through the synthesis of cytokines and growth factors. *In vitro*, hyperglycaemia stimulates the release of IL-6 resulting in human mesangial and tubular cell proliferation, hypertrophy, and diabetic nephropathy via enhanced JAK/STAT signalling [39]. Hyperglycaemia also increases expression of ICAM-1, E-selectin, and VCAM-1 in human umbilical vein ECs (HUVECs) independently of an alternative proinflammatory stimulus, tumour necrosis factor alpha (TNF-*α*) [40, 41]. Sustained hyperglycaemia increased rat aortic SMC proliferation through downregulation of protein kinase C *β*II (PKC *β*II) [42]. Hyperglycaemic responses may differ according to species, however, as a study using human SV-SMC observed no effect of elevated glucose levels (25 mM) on proliferation [23]. In summary, the above studies suggest that hyperglycaemia may contribute to vascular inflammation and cell migration and proliferation, all of which are key events in the development of NIH in SVGF.

Hyperinsulinaemia can also induce EC and SMC dysfunction through abnormal activation of key signalling pathways. During insulin resistance, the phosphatidylinositide-3-kinase (PI3K) signalling pathway that controls the metabolic effects of insulin activation is impaired, whilst activation of the proinflammatory, mitogenic extracellular signal-regulated kinase 1/2 (ERK1/2) pathway is enhanced. High concentrations of insulin (100 nM) can stimulate proliferation of human fragenicular SMCs cultured from T2DM patients [43] and human SV-SMCs via the sterol regulatory element-binding transcription factor 1 (SREBF1) pathway [44], whilst human SV-ECs are unaffected. High insulin (100 nM) concentrations have also been shown to increase migration of bovine aortic SMCs through increased activation of ERK1/2 pathway. This study also observed impaired PI3K pathway [45]. This is contrary to the study performed in human SV-SMCs where hyperinsulinaemia (100 nM) increased cell migration independently of any PI3K pathway dysfunction [23]. However, the combination of increased SV-SMC proliferation and migration was enough to lead to a significant augmentation of NIH development *ex vivo* [44]. Together, these studies suggest that compensatory hyperinsulinaemia associated with insulin resistance has stimulatory effects in vascular cell migration and proliferation that may be detrimental (Figure 1).

### 2.2. Inflammation, NIH, and T2DM

Chronic inflammation is a consequence of T2DM and an important pathophysiological process responsible for NIH and SVGF [15, 46, 47]. Conditions prevalent in T2DM evoke the production of chemokines, proinflammatory cytokines, and adhesion molecules [48], all of which can modulate cell behaviour. Low-grade chronic inflammation found in T2DM is centrally controlled by elevation of proinflammatory cytokines such as interleukin-1 (IL-1), TNF-*α*, and IL-6, mediating chronic tissue injury. There is also growing evidence that anti-TNF-*α* biological drugs used to treat rheumatoid arthritis (RA) can improve insulin sensitivity and reduce insulin resistance in RA patients, raising the possibility that targeting proinflammatory pathways may also have therapeutic benefits in T2DM patients [49]. This review will focus on IL-6, the plasma concentration of which is approximately 1pg/ml in healthy individuals and increases 2-3 folds in T2DM [13, 47, 50–53].

The role of IL-6 in vascular inflammation and NIH pathology is supported by multiple *in vivo* and *in vitro* studies, with high levels of IL-6 in patients undergoing CABG correlating with graft occlusion and cardiovascular events such as stroke, myocardial infarction, and repeat CABG [28, 54, 55]. The potential utility of IL-6 measurements for predicting specific outcomes was shown by the finding that preoperative serum levels of IL-6 exceeding 3.8 pg/ml were associated with a significantly higher risk of vein graft occlusion and late cardiovascular events [54]. In HUVECs and human aortic endothelial cells (HAECs), IL-6 can induce expression of ICAM-1 and MCP-1, which are involved in monocyte recruitment to the endothelium [56, 57]. IL-6 also triggers the release of vascular endothelial growth factor (VEGF) in HAECs thereby increasing angiogenesis [58], while stimulation of rat thoracic aorta SMCs with IL-6 and TNF-*α* results in increased expression of MCP-1 and ICAM-1 [59, 60]. In addition to its proinflammatory effects, IL-6 has been shown to increase rat thoracic aorta SMC migration and proliferation [59, 60]. This was corroborated in a study using human carotid and mice aortic SMCs, whereby IL-6 increased STAT3 activation and proliferation of SMCs and augmented MCP-1 and ICAM-1 expression (Figure 1) [61]. Whilst there are limited *in vivo* and *ex vivo* studies which build upon these *in vitro* findings, significantly higher levels of IL-6 and other inflammatory mediators (ICAM-1, MCP-1, and TNF-*α*) have been found in NIH lesions versus healthy vein in a rat coronary artery bypass graft model [60]. Given the evidence demonstrating a critical role of IL-6 in endothelial inflammation, SMC migration and proliferation, this pro-inflammatory cytokine represents a potential therapeutic target for treatment of NIH.

## 3. IL-6/JAK/STAT Pathway

Given the evidence presented above for elevated IL-6 in T2DM patients, the correlation of high circulating IL-6 with poor revascularisation outcomes, and the known vascular cell aberrancies in T2DM, it is pertinent to review the IL-6 signalling pathway. This consists of three major parts: receptor activation, activation of the JAK/STAT pathway, and termination of the cascade via suppressor of cytokine signalling 3 (SOCS3).

### 3.1. The IL-6 Receptor and Classical versus Trans-Signalling

IL-6 is a 21–28 kDa glycosylated protein comprising 184 amino acids. Cellular responses to IL-6 are mediated through a receptor complex consisting of IL-6R*α* (CD126) and glycoprotein 130 (gp130) [62]. IL-6 belongs to a family of cytokines which also include oncostatin M (OSM), interleukin-11 (IL-11), cardiotrophin-1 (CT-1), leukaemia inhibitory factor (LIF), and ciliary neurotrophic factor (CNTF) [63]. Binding of IL-6 family cytokines to their receptor complexes involves gp130 homodimerisation (in the case of IL-6 and IL-11) or heterodimerisation with LIF-R or OSM-R (in the cases of LIF-*β*, OSM-*α*, CNTF, or CT-1-*α*) to activate JAK/STAT signalling [63]. Importantly, IL-6 signalling is implicated in mediating the vascular complications of diabetes and the development of NIH and accelerated atherosclerosis responsible for SVGF [47, 52, 60].

**Figure 2:**
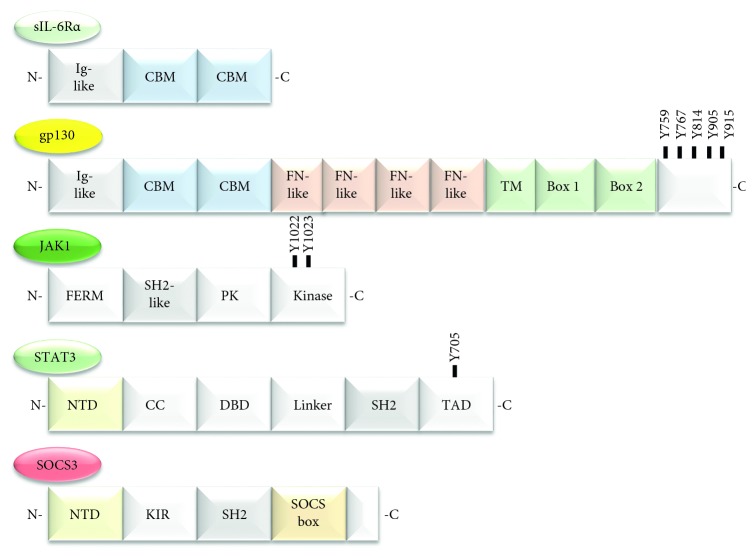
Protein domains in the IL-6 signalling pathway. sIL-6R*α* comprises an Ig-like domain and two cytokine-binding modules (CBM). gp130 also has these three domains at the N-terminus, and these are followed by four fibronectin-like domains (FN-like), a transmembrane domain (TM), and intracellular box 1 and box 2 domains required for JAK binding and signalling. JAK contains a band 4.1, ezrin, radixin, moesin domain (FERM), an SH2-like domain, a pseudokinase domain (PK), and a kinase domain. STAT3 contains an N-terminal domain (NTD), coiled coil domain (CC), a DNA-binding domain (DBD), a short linker region, and then an SH2 domain. For SOCS3, this contains an NTD, kinase inhibitory region (KIR) which inhibits JAK, SH2 domain, and SOCS box responsible for recruiting components of the E3 ubiquitin ligase complex. Key phosphorylated tyrosine residues (Y) involved in signalling protein activation are indicated.

**Figure 3:**
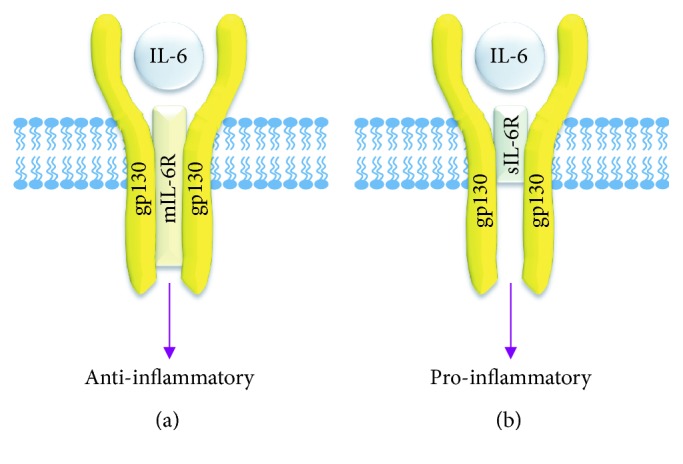
IL-6 classical and trans-signalling. (a) In classical signalling, IL-6 binds to a membrane-bound IL-6 receptor molecule in addition to two flanking gp130 molecules. Signalling through this pathway mediates anti-inflammatory signalling and is prevalent in blood cells and hepatocytes. (b) Trans-signalling occurs on the majority of other cell types, including cells of the vascular walls. Here, a soluble IL-6 receptor molecule is utilised in combination with two gp130 molecules. This truncated, soluble IL-6 receptor is responsible for many of the proinflammatory actions of IL-6 in disease.

The structure of IL-6 consists of four long and straight *α*-helices (helix A, B, C, and D) with three receptor-binding sites [64]. IL-6 R*α* is an 80 kDa glycoprotein consisting of an extracellular region made up of an N terminal Ig-like domain which stabilises the receptor and two CBMs which are required for IL-6 binding linked to a short intracellular domain [65] (Figure 2). Gp130 has a similar structure as IL-6R*α* with additional four extracellular domains termed IgG-like fibronectin-like domains and an intracellular domain containing box 1 and box 2 regions necessary for signalling [66]. The receptor also exists in two forms: membrane bound (mIL-6R*α*) and soluble (sIL-6R*α*), the latter of which does not contain an intracellular domain (Figure 3).

IL-6 binds to a specific transmembrane IL-6R*α* expressed on the surface of target cells, and the IL-6/IL-6R*α* complex binds two molecules of transmembrane protein gp130 to activate intracellular signalling pathways including JAK/STAT, ERK1/2, and PI3K [62, 67]. Gp130 is ubiquitously and constitutively expressed [68]. In contrast, mIL-6R*α* expression is restricted to monocytes, hepatocytes, neutrophils, and some B- and T-cell subsets [69]. When the cell expresses both gp130 and IL-6R*α*, this is referred to as classical signalling [70, 71]. Vascular EC and SMC lack membrane localised IL-6R*α* but are still targets of IL-6 signalling because circulating sIL-6R*α* can form a complex with IL-6 and associate with gp130 to activate signalling during inflammation. This phenomenon is termed trans-signalling and increases the spectrum of target cells that can respond to IL-6 (Figure 3). sIL-6R*α* is translated from alternatively spliced IL-6R*α* mRNA [72]. It is also generated from proteolytic processing of mIL-6R*α* by ADAM10, ADAM17, and meprin metalloproteinases [73, 74]. Due to the different receptor complexes for IL-6 described above, this cytokine mediates both anti- and proinflammatory responses though classical and trans-signalling, respectively, contributing to disease progression [71]. Whilst serum levels of sIL-6R*α* are the same for T2DM and NDM patients, the elevation in circulating IL-6 means that plasma levels of the IL-6/sIL-6R*α* complex are increased in diabetic patients [75], raising the likelihood of the signalling pathway being inappropriately activated.

### 3.2. JAK/STAT Signalling

**Figure 4:**
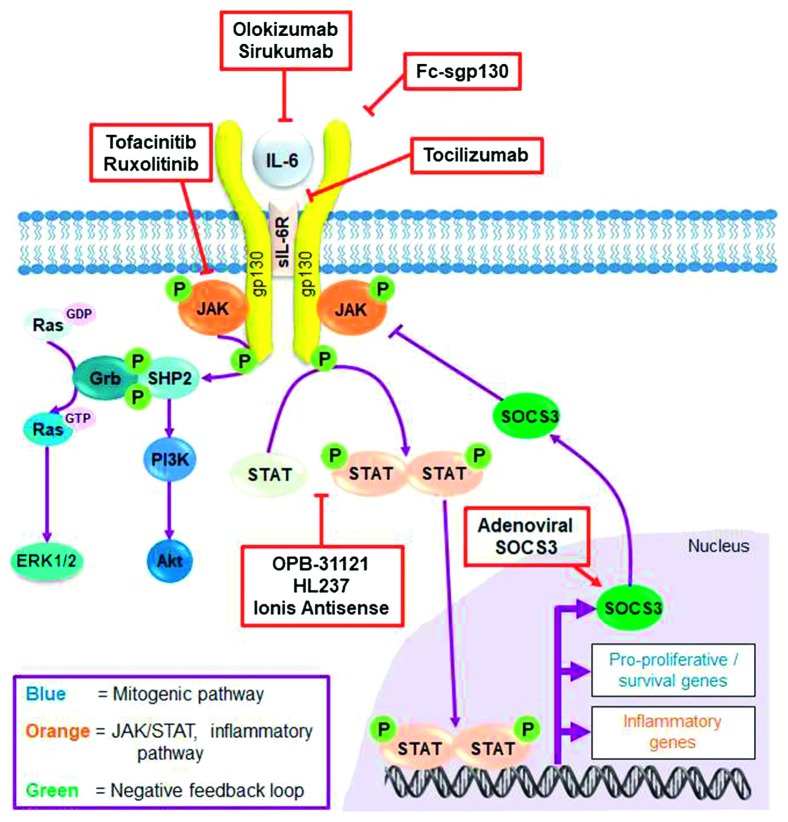
The IL-6–JAK/STAT–SOCS3 signalling pathway. The principal signalling pathway triggered by IL-6 is the JAK/STAT pathway (orange). JAK phosphorylates gp130 leading to phosphorylation and dimerisation of STAT, which translocates to the nucleus to induce expression of prosurvival and proinflammatory genes. It also induces expression of SOCS3 as a negative feedback loop to switch off the signalling pathway (green). SOCS3 forms part of the E3 ubiquitin ligase complex to ubiquitinate JAK, leading to proteasomal degradation and termination of the signalling cascade. JAK can also act independently of STAT to trigger additional mitogenic and growth-promoting effects via the PI3K/Akt and ERK1/2 pathways (blue). Sites of action of specific therapeutic inhibitors are indicated in red boxes.

The JAK/STAT pathway is the principal signalling pathway triggered by IL-6, whilst it also triggers activation of ERK1/2 and PI3K, these are downstream of JAK and reliant on the recruitment of SH2 domain containing protein tyrosine phosphatase 2 (SHP-2) [62] (Figure 4). Alterations in JAK/STAT signalling have been implicated in various complications of T2DM [76].

Binding of IL-6 to the receptor complex induces conformational changes in the cytoplasmic domain of gp130 causing JAKs activation [77]. There are four JAKs (JAK 1–3 and Tyk2 (tyrosine kinase 2)). JAK proteins have seven Janus homology (JH) domains [78]. The JH1 domain at the C terminus has an intrinsic tyrosine kinase activity and includes a conserved double Tyr (YY) motif involved in trans-phosphorylation. The JH2 domain has Ser/Tyr kinase activity which is responsible for regulating the activity of the JH1 domain [79]. JH3-5 comprise an SH2-like domain, and JH6-JH7 at the N terminus comprises a FERM domain (band 4.1, ezrin, radixin, moesin), which mediates JAK interaction with gp130 (box1/2 region) [66] (Figure 2). JAK activation involves trans-phosphorylation at the conserved double tyrosine motif of the JH1 activation loop, causing the activation loop to shift away from the catalytic site to allow access of protein substrates and ATP [80]. The activated JAK in turn phosphorylates the four most distal membrane tyrosine receptor motifs (Tyr^767^, Tyr^814^, Tyr^905^, and Tyr^915^) within the cytoplasmic tail of gp130 [70]. Subsequently, STAT3 and to a lesser extent STAT1 are recruited via their SH2 domains to the phosphorylated tyrosine motifs of gp130 [70] (Figure 4).

There are seven STAT isoforms (STAT1-4, 5a, 5b, and 6). STATs have a basic five-domain structure consisting of a coiled coil domain at the N terminus preceded by a DNA-binding domain, a linker domain (which links the DNA-binding domain to an SH2 domain), an SH2 domain, and a transactivation domain at the C terminus (Figure 2). STAT activation requires phosphorylation at Tyr^701^ for STAT1 and Tyr^705^ for STAT3 in the C terminal trans-activation domain [81]. The activated STATs then dimerise and translocate to the nucleus where they bind to two types of response elements: interferon-stimulated response elements (ISREs) and gamma-activated sites (GAS). The ISRE appears to be restricted to IFN signalling, whereas the GAS, including sis-inducible element (SIE), acute-phase response element (APRE), and other GAS-like sequences are present in promoters of genes such as the acute-phase proteins that are well-defined STAT targets [81]. The phospho-Tyr^759^ motif of gp130 is involved in recruitment of the key negative modulator of IL-6 signalling, SOCS3. Tyr^759^ is also necessary for recruitment of SHP-2 which leads to activation of ERK1/2 and PI3K pathways. However, IL-6 signals through JAK/STAT3 to mediate the majority of its physiological effects [69].

### 3.3. Suppressor of Cytokine Signalling 3 (SOCS3)

SOCS genes encode a family of 8 intracellular proteins (SOCS 1–7 and cytokine inducible SH2 (CIS)) with SOCS1 and SOCS3 being the most well studied [82]. SOCS proteins are generally expressed at low concentrations under basal conditions but are rapidly induced by IL-6 stimulation. As a result, induced SOCS3 can block IL-6/JAK/STAT activity to form a classic negative feedback loop. Other stimuli that induce SOCS3 include cyclic AMP [83], erythropoietin (EPO), growth hormone, insulin, granulocyte macrophage colony stimulating factor (GM-CSF), prolactin, leptin, interferons (IFNs), IL-2, and IL-9 [82]. Whilst they are undoubtedly beneficial in dampening inflammatory responses, they have also been implicated in impairing glucose tolerance and thus have a complex relationship with T2DM [84].

All SOCS proteins have a similar structure that include a conserved C-terminal SOCS box domain (which is essential for assembly of a functional E3 ubiquitin ligase complex required for ubiquitylation of target proteins) and a conserved central SH2 domain which is essential for binding to Tyr-phosphorylated motifs in target proteins [85]. Another component of SH2 domain is the PEST sequence (Pro-Glu-Ser-Thr-rich) which controls the biological half-life of SOCS3 [86, 87]. SOCS1 and SOCS3 also contain a kinase inhibitory region (KIR) in the N terminal region of the protein which is responsible for inhibiting JAK activity [88] (Figure 2).

SOCS3 inhibits IL-6-induced JAK/STAT activation through direct inhibition of JAK kinase activity, preventing both substrate and ATP binding. SOCS3 uses its SH2 domain to bind phosphorylated Tyr^759^ on the cytoplasmic tail of gp130 and uses the reverse face of the SH2 domain to bind to the JAK kinase domain around a glycine-glutamine-methionine (GQM) sequence in the JH1 domain [89]. The simultaneous binding of SOCS3 to both the cytokine receptor and JAK is thought to explain SOCS3 selectivity in inhibiting certain cytokines that signal through gp130. SOCS3 targets a unique GQM motif found in JAK1, JAK2, and Tyk2, but not JAK3 [90]. Once bound, the SOCS box domain in SOCS acts as a scaffold for assembly of a multisubunit E3 ubiquitin ligase complex to target JAKs for ubiquitylation and proteasome-dependent degradation [91], thus terminating the JAK signalling cascade. The E3 ubiquitin ligase complex is made up of cullin5, elongin B/C, and RING box-2 (Rbx2) and SOCS box [92]. The formation of an E3 ubiquitin ligase complex is the final step in attachment of ubiquitin to SOCS substrate; this complex adds Lys48-linked chains of ubiquitin (a highly conserved 76 amino acid protein) to Lys residues on target proteins (e.g. JAKs), which serves as a marker for recognition and degradation by the proteasome [93].

The effects of SOCS3 are limited by its short biological half-life which is cell-dependent, for example, 30 minutes in mouse-derived Ba/F3 hematopoietic cells [94] and 2 hours in monkey-derived COS-7 cells [95]. SOCS3 protein stability is regulated by both proteasomal and nonproteasomal degradation pathways. Several studies have shown that nonproteasomal degradation involves the PEST sequence, a 35 amino acid unstructured Pro-Glu-Ser-Thr-rich sequence found in the SH2 domain of SOCS3 which does not affect binding to Tyr-phosphorylated cytokine receptor. This sequence has a destabilising role in SOCS3 as removal of this in HEK293 T cells increased SOCS3 stability [96]. Another mechanism controlling stability of SOCS3 is phosphorylation of the SOCS box at Tyr^204^ and Tyr^221^ which reportedly enhanced proteasome-dependent SOCS3 degradation in COS-7 cells [95]. Disruption of the interaction between elongin B and C with the SOCS box destabilised SOCS3 protein expression [95]. Studies in murine Ba/F3 hematopoietic cells identified that the N-terminal eleven amino acids in SOCS3 were crucial for destabilisation. In particular, Lys^6^ was identified as a key residue that regulates SOCS3 ubiquitylation and stability [94].

## 4. Opportunities for Therapeutic Applications

It is clear that atherosclerosis, NIH, and SVGF are substantial clinical issues, which can be exacerbated in T2DM. Currently, the only pharmacological treatment available for graft restenosis is antiplatelet therapy [97]. However, this does not consistently inhibit the development of NIH [98]. Furthermore, some drug-eluting stents used during PCI inhibit re-endothelialisation and so can increase the risk of thrombosis [99, 100]. For example, in a five-year randomised clinical trial examining sirolimus-eluting stents in 1,748 DM patients, probable stent thrombosis after 1 year was doubled compared to control bare-metal stents group [101]. Therefore, there is a need for agents that selectively inhibit NIH without altering EC migration and proliferation.

Many promising approaches to target IL-6/JAK/STAT signalling pathway have been explored; however, they are not without difficulties. These include neutralising antibodies which target IL-6 or IL-6R*α*, JAK inhibitors, and STAT inhibitors.

### 4.1. IL-6/IL-6R Blockade

As the IL-6/sIL-6R*α* pathway activates the proinflammatory trans-signalling in EC and SMC, studies have examined whether blockade of this pathway is a feasible clinical treatment. An *in vitro* coculture model of human EC and SMC was utilised to examine the effects of IL-6 signalling pathway inhibitors, namely, sirukumab (anti-IL-6 antibody), tocilizumab (anti-IL-6R*α* antibody), and tofacitinib (JAK inhibitor) in vascular inflammation under atherogenic conditions. Cells were cultured under proatherogenic flow conditions in the presence of TNF-*α* and oxLDL and then stimulated with sIL-6R*α*. Proinflammatory responses were dampened by sirukumab and tocilizumab, with a significant reduction in adhesion molecule gene expression and an increased contractile SMC phenotype. Importantly, tofacinitib was less effective in modulating SMC phenotype and inflammation, suggesting that inhibiting the initial pathway trigger may have greater anti-inflammatory and vascular protective efficacies versus targeting of downstream signalling molecules [102].

Tocilizumab is a humanised monoclonal antibody against both membrane-bound and sIL-6R*α* [103]. This agent was approved by the Food and Drug Administration (FDA) in 2010 for treatment of RA and in 2011 for treatment of systemic juvenile idiopathic arthritis. It is given by injection intravenously and/or subcutaneously [104] and has undergone limited evaluation for efficacy in CVD. A recently completed phase II clinical trial examined its ability to reduce acute vascular inflammation in nonS/T-elevation myocardial infarction (NSTEMI) patients; however, the anticipated cardioprotective effect was not clear-cut, with tocilizumab causing a selective upregulation of inflammatory mediators interferon gamma-inducible protein 10 (IP-10) and macrophage inflammatory protein-1*β* (MIP-1*β*) [105]. Given that many patients undergoing CVD interventions already suffer from underlying chronic inflammation observed in conditions such as T2DM, this potential proinflammatory effect may limit its usefulness in the clinic. Furthermore, this clinical trial was not able to fully recapitulate the effects of tocilizumab that were evident *in vitro* [105].

There are also neutralising antibodies against IL-6 in clinical trials which include olokizumab and sirukumab for rheumatoid arthritis and cancer, but these are not evaluating cardiovascular outcomes. However, the main limitation of neutralising antibodies targeting either IL-6 or IL-6R*α* is that they inhibit classical and trans-signalling nonselectively [106]. Efforts to selectively block proinflammatory trans-signalling that has been implicated in CVD has led to the development of a recombinant-engineered Fc-sgp130 fusion protein. Normally, a combination of sIL-6R*α* and sgp130 results in antagonistic activity, and therefore, a complex of IL-6, sIL6-R*α*, and Fc-sgp130 fusion protein reduces IL-6 bioavailability [107]. This approach is currently in phase II clinical trials for management of alcoholic liver disease and chronic hepatitis C virus infection [108].

### 4.2. JAK/STAT Inhibition

Low molecular weight orally bioavailable JAK inhibitors are being explored in the clinic for specific indications. Tofacitinib is a first-generation nonselective JAK inhibitor with a pyrrol (2, 3-d) pyrimidine derivative which was approved by the FDA in 2012 for treatment of RA [104]. It inhibits JAK1, JAK2, and JAK3 proteins with low nM potencies (IC_50_ = 0.16 nM, 0.58 nM and 1.6 nM respectively) with a 30-fold decrease in potency at Tyk2 (IC_50_ = 4.8 nM) compared to JAK1 [109]. Although phase III clinical trials for Tofacitinib showed an increased risk of cellulitis, herpes zoster, a decrease in neutropenia and increased levels of low- and high-density lipoproteins, this drug is still used due to lack of novel agents available to block the deleterious effects of IL-6 [110]. There are no cardiovascular risks reportedly associated with tofacitinib [111, 112], although trials examining specific benefits under pathological cardiovascular conditions are yet to be reported. Ruxolitinib is another orally bioavailable first-generation JAK inhibitor. Ruxolitinib has high potency at sub-nM range for JAK1 (IC_50_ = 0.09 nM) and JAK2 (IC_50_ = 0.036 nM). This drug was approved by the FDA in 2011 for management of polycythemia vera and myelofibrosis [113]. Second-generation JAK inhibitors and also STAT inhibitors are currently in clinical trials (reviewed by [114, 115]), although these are at an early stage.

Accelerated JAK/STAT activation has been implicated in pathologies such as restenosis in PCI and SVGF, atherosclerosis, and inflammatory disease [70, 116]. Activation of STAT3 by JAK1 and JAK2 particularly has been found to regulate SMC migration and proliferation [117, 118]. STAT3 leads to transcription of IL-6-responsive genes such as cyclin D1 (involved in SMC proliferation), survivin (an antiapoptotic protein that reduces SMC death), VEGF (responsible for angiogenesis), and MCP-1 leading to leukocyte infiltration [56, 116, 119–121]. As such, STAT3 is considered to contribute to the development of NIH and SVGF, and accordingly, a considerable body of research has tried to identify selective therapeutic inhibitors of STAT3. These have principally been as treatments for malignancy, inflammation, and autoimmune diseases. Small molecules targeting SH2-binding domains (necessary for STAT3 binding to the receptor and STAT3 dimerisation) and STAT-binding site oligonucleotide decoys are strategies that have been explored to inhibit STAT3 activation in clinical trials (reviewed by [115]). The small molecule STAT3 inhibitor OPB-31121 was tested in a clinical trial for advanced solid tumours; however, limited efficacy and unfavourable side effects including peripheral neuropathy were reported [122], which would limit its use for CVD in the T2DM population. Other STAT3 inhibitors such as HL237 and Ionis antisense therapy are currently undergoing clinical trials for autoimmune diseases and cancer (ClinicalTrials.gov ID NCT03278470 and NCT01563302, respectively). However, these are at an early stage and whether cardiovascular risk or benefit will be measured is unknown.

It is important to note that both JAKs and STATs are coupled to a number of other cytokines [71] important for a range of physiological functions. For example, JAKs also couple to IL-2R [123]. IL-2 is required for immune T-cell activation [124] and thus JAK inhibitors could be considered immunosuppressive. Whilst this is beneficial for patients with a hyperactive immune system (e.g., RA), it may not be appropriate for CVD and SVGF due to the increased risk of infections in an already vulnerable patient population. Therefore, the downstream signalling of IL-6-dependent gp130/JAK/STAT signalling (e.g., SOCS3) could represent a more specific target for reducing SVGF.

### 4.3. SOCS3 Upregulation

The protective role of SOCS3 in limiting vascular inflammation is well established [70]. A central component of this is the ability of SOCS3 to limit IL-6 signalling [120] and thus protect against NIH and CABG restenosis, and other inflammatory diseases such as atherosclerosis [61]. Mice lacking SOCS3 develop a chronic inflammatory response induced by IL-1*β*, whereas mice lacking both IL-6 and SOCS3 or IL-6 only were protected from this inflammatory response, demonstrating the efficacy of IL-6 blockade by SOCS3 *in vivo* [125].

Immunohistochemical analysis of balloon-injured porcine coronary arteries indicated loss of SOCS3 in NIH as compared to media [126]. However, SOCS3 has been shown to be upregulated in human carotid atherosclerotic plaques and colocalised with markers for macrophages and SMCs [61]. This was thought to be a compensatory mechanism that was insufficient to inhibit molecular processes leading to development of vascular remodelling. The same study found that deletion of SOCS3 by small interfering (si) siRNA in Apo E^−/−^ mice resulted in a significant increase in atherosclerosis lesion size in the aorta as compared to controls (confirming the protective role of SOCS3) and significantly increased SMC and macrophage proliferation *in vitro* [61]. Conversely, overexpressing SOCS3 via adenovirus transduction in a rat model of vein grafting had a beneficial effect with a reduction in SMC migration, proliferation, and inflammatory markers (IL-1*β*, IL-6, MCP-1, ICAM-1, and TNF-*α*), accompanied by a reduction in NIH [60]. Taken together, these studies indicate that lack of SOCS3 is a major contributor to enhanced signalling in CVD causing NIH and restenosis, and its induction may indeed be of benefit.

One possible mechanism by which SOCS3 reduces cell migration is by promoting the ubiquitylation and subsequent proteasomal degradation of focal adhesion kinase 1 (FAK 1) [127]. FAK 1 is a nonreceptor protein-tyrosine kinase involved in integrin-mediated adhesion and regulates cell motility [128]. These studies all highlight the beneficial role of SOCS3 in limiting inflammation and vessel remodelling; however, the short half-life of SOCS3 *in vivo* limits its therapeutic potential.

Different approaches targeting SOCS3 to increase its efficacy and function have been tested. Therapeutic trials using adenoviral delivery of SOCS3 have been reported in experimental arthritis. Injection into the ankle joints of mice with collagen or antigen-induced arthritis significantly inhibited the severity of arthritis and joint inflammation [129], showing that the approach is viable (at least in animal models). Adenoviruses have attracted much attention in gene therapy because they mediate transient gene expression and can be made replication incompetent, hence no expression of viral proteins after transduction. More recently, adenoviruses encoding TIMP3 have been shown to be effective in blocking short- (28 days) and long-term (3 months) NIH in pig models of vein graft failure [33, 36, 37]. Gene therapy is particularly an attractive option for the treatment on graft restenosis as it can specifically target local inflammation, migration, and proliferation. Furthermore, there is a window where the blood vessel is outside the body before implantation during which time it can be treated. Replenishment of SOCS proteins by exogenous administration of recombinant, cell-penetrating versions of SOCS3 has also been shown to reduce inflammation *in vivo*. SV blood plasma levels of IL-6 and TNF-*α* were measured before and after treatment with cell-penetrating SOCS3 from C3H/HeJ mice with levels significantly inhibited after treatment [130]. Furthermore, endogenous SOCS3 can be increased in response to cyclic AMP elevation to inhibit EC inflammation *in vitro* [83].

It is clear from these studies that restoration of SOCS3 can be achieved and has great potential in limiting vascular inflammation and SMC migration and proliferation. Thus, SOCS3 is a promising therapeutic target for reducing NIH and SVGF. Indeed, it has been proposed to be effective in ameliorating some of the microvascular complications of diabetes, namely, nephropathy in *in vitro* and *in vivo* studies in rat [39]. Whether this could be translated into the macrovasculature and human disease remains to be seen.

## 5. Conclusion

Deregulation of the IL-6–JAK/STAT–SOCS3 pathway is implicated in the development of CVD, in particular in SVGF and NIH. Given that both SVGF and NIH are more common in the T2DM population, and the alarming rate at which T2DM is increasing globally, there is an urgent need for the development of new therapeutic strategies to counteract CVD. Development, testing, and approving of new therapies is a notoriously time-consuming process, and so it may be necessary to repurpose existing therapeutics for other disorders in the cardiovascular field. Accordingly, the potential effects of existing therapies targeting this pathway on cardiovascular outcomes are listed in Table 1.

With the off-target effects inherent to IL-6 inhibition (blocking both classical and trans-signalling) and JAK/STAT inhibitors, it is likely that new therapeutics will evolve from SOCS3. The anti-inflammatory, antimigratory, and anti-proliferative effects of SOCS3 *in vitro* and *in vivo* highlight its potential as a therapeutic option in NIH and SVGF. It will be exciting to see whether this can successfully translate into the clinic over the coming years.

## Figures and Tables

**Table 1:** Selected IL-6–JAK/STAT–SOCS3 modulators and their impact on cardiovascular disease.

Agent	Mode of action	Type of study	Outcome
Sirukumab	IL-6-blocking antibody	*In vitro*	↓ Adhesion molecule expression ↑ SMC contractile phenotype
Fc-sgp130	IL-6 trans-signalling inhibitor	In clinic	Awaiting outcome
Tocilizumab	IL-6R*α*-blocking antibody to inhibit classical and trans-signalling	*In vitro* In clinic	↓ Adhesion molecule expression ↑ SMC contractile phenotype Approved for rheumatoid arthritis and systemic juvenile idiopathic arthritis ↑ IP-10 and MIP-1*β* in acute phase STEMI
Tofacitinib	JAK inhibitor	In clinic	Approved for rheumatoid arthritis No effect on cardiovascular risk
Ruxolitinib	JAK inhibitor	In clinic	Approved for polycythemia vera and myelofibrosis no studies reporting on cardiovascular system
OBP-31121	STAT3 inhibitor	Clinical trial	↑ Peripheral neuropathy no studies reporting on the cardiovascular system
Adenoviral SOCS3	Increases SOCS3-mediated inhibition of signalling	*In vivo*	↓ SMC inflammation, migration, and proliferation ↓ NIH

Selected existing therapies targeting the IL-6–JAK/STAT–SOCS3 signalling pathway. Please see text for detail and references.

## Abbreviations

APRE: Acute-phase response element
CABG: Coronary artery bypass graft
CAD: Coronary artery disease
CBM: Cytokine-binding module
CIS: Cytokine-inducible SH2 protein
CNTF: Ciliary neurotrophic factor
CT-1: Cardiotrophin-1
CVD: Cardiovascular disease
DM: Diabetes mellitus
EC: Endothelial cell
EPAC: Exchange protein directly activated by cAMP
EPO: Erythropoietin
ERK1/2: Extracellular signal-regulated kinase 1/2
FAK 1: Focal adhesion kinase 1
FDA: Food and Drug Administration
FERM: Band 4.1, ezrin, radixin, moesin
GAS: Gamma-activated sites
GM-CSF: Granulocyte macrophage colony stimulating factor
gp130: Glycoprotein 130
HAEC: Human aortic endothelial cell
HSV: Human saphenous vein
HUVEC: Human umbilical vein endothelial cell
ICAM-1: Intercellular adhesion molecule-1
IFN: Interferon
IL: Interleukin
IMA: Internal mammary artery
IP-10: Interferon gamma-inducible protein 10
JAK: Janus kinase
JH: JAK homology domain
KIR: Kinase inhibitory region
LIF: Leukaemia inhibitory factor
MCP-1: Monocyte chemoattractant protein-1
mIL-6R*α*: Membrane-bound interleukin-6 receptor *α*
MIP-1*β*: Macrophage inflammatory protein-1 *β*
MMPs: Matrix metalloproteinases
NDM: Nondiabetes mellitus
NIH: Neointimal hyperplasia
NSTEMI: Non-S/T-elevation myocardial infarction
OSM: Oncostatin M
PCI: Percutaneous coronary intervention
PECAM-1: Platelet endothelial cell adhesion molecule-1
PI3K: Phosphatidylinositol-3 kinase
RA: Rheumatoid arthritis
Rbx: Ring box 2
SHP-2: SH2 domain containing protein phosphatase 2
si: Small interfering
SIE: Sis-inducible element
sIL-6R*α*: Soluble interleukin-6 receptor *α*
SMC: Smooth muscle cell
SOCS3: Suppressor of cytokine signalling 3
SREBF1: Sterol regulatory element binding transcription factor 1
STAT: Signal transducer and activator of transcription
SVGF: Saphenous vein graft failure
T2DM: Type 2 diabetes mellitus
TIMP: Tissue inhibitor of MMPs
TNF-*α*: Tumour necrosis factor *α*
Tyk2: Tyrosine kinase 2
VCAM-1: Vascular cell adhesion molecule-1
VEGF: Vascular endothelial growth factor
vWF: Von Willebrand factor.
